# The behavioral response of *Lasioderma serricorne* (Coleoptera: Anobiidae) to citronellal, citral, and rutin

**DOI:** 10.1186/s40064-016-2553-2

**Published:** 2016-06-21

**Authors:** Jianhua Lü, Shuli Liu

**Affiliations:** Province Key Laboratory of Transformation and Utilization of Cereal Resource, School of Food Science and Technology, Henan University of Technology, Lianhua Street, Zhengzhou High-Tech Development Zone, Zhengzhou, 450001 Henan China

**Keywords:** Behavioral response, *Lasioderma serricorne*, Citronellal, Citral, Rutin

## Abstract

The behavioral response of *Lasioderma serricorne* adults to citronellal, citral, and rutin was investigated by using the area preference method. The *L. serricorne* adults were exposed to citronellal, and citral at the rate of 1:10, 1:50, 1:100 and 1:1000 (citronellal: ethanol, v/v) for 1, 2, 12 and 24 h, to rutin at the rate of 10, 30 and 90 g/m^2^ for 1, 2, 12 and 24 h, respectively. The citronellal and citral had attractive activity at the low rates and repellent potential at the high rates. The highest behavioral response values of *L. serricorne* adults to citronellal and citral were −88.89 % at the rate of 1:100 and 100.00 % at the rate of 1:50 respectively. Rutin had strong repellent effectiveness on *L. serricorne* adults, which significantly increased with increasing rates with the highest behavioral response values 100.00 % at the rate of 90 g/m^2^ after 12 h exposure. These data suggest that the citronellal, citral, and rutin have great potential for preventing stored products from *L. serricorne* infestation.

## Background

The cigarette beetle, *Lasioderma serricorne* (Fabricius) (Coleoptera: Anobiidae) ranks as one of the most serious pests of stored products in the world (Kim et al. 2003). The cigarette beetle was first found in the tomb of Tutankhamun (Alfieri 1931) and Rameses II (Steffan 1982). The earliest records of the cigarette beetle associated with tobacco appeared in France in 1848 (Runner 1919) and in the United States of America in 1886 (Tenhet and Bare 1951). The *L. serricorne* larvae usually cause damage by eating tobacco and penetrate deep into tobacco mass, resulting in small round holes in the tobacco and its products.

Phosphine has been used for the control of *L. serricorne* population since 1950s. However, its repeated and intensive use has resulted in serious negative issues including insecticide resistance, insecticide residue, insect resurgence, and lethal effects on non-target organisms (Jovanović et al. 2007). Development and application of environment-friendly control strategies and integrated pest management (IPM) systems have recently been considered to be the only sustainable solution to combat the increasing insecticide-resistant insects (Kim et al. 2003).

Behavioral manipulation is an important insect control method based on insect behavioral responses to special environmental factors. Particularly, the repellents and attractants have been often applied to manipulate insect behaviors, which can effectively prevent crops and stored products from insect infestation. Most of stored product insects, such as *Tribolium castaneum* Herbst (Coleoptera: Tenebrionidae) (Campbell 2012), *Tribolium confusum* (Coleoptera: Tenebrionidae) (Athanassiou et al. 2006), *Sitophilus granarius* (L.) (Coleoptera, Dryophthoridae) (Germinara et al. 2012a, b), *Sitophilus zeamais* Motschulsky (Coleoptera: Curculionidae) (Trematerra et al. 2013), *Sitophilus oryzae* (Coleoptera: Curculionidae) (Kumar et al. 2004), *Ahasverus advena* (Waltl) (Coleoptera: Cucujidae) (Wakefield et al. 2005), and *Oryzaephilus surinamensis* (Coleoptera: Silvanidae) (Mowery et al. 2004) respond preferentially to the volatiles of cereal grains, processed products, or pheromone. Some compounds have been verified to have potent potential as attractants or repellents for practical application.

Citronellal is a monoterpenoid with distinctive lemon scent. Some researches have showed that citronellal has insect repellent properties, especially against mosquitoes (Kim et al. 2005). Citral has a strong sweet lemon odor with strong antimicrobial qualities (Onawunmi 1989), repellent effects against *Callosobruchus maculatus* (Ke et al. 1992), and pheromonal effect (Robacker and Hendry 1977). Rutin is one of the phenolic compounds found in many plants, including the fruits and rinds of peaches *Prunus persica* Linn and apples *Serica orientalis* Motschulsky, especially the tartary buckwheat plant *Fagopyrum tataricum* Gaertn belonging to family Polygonaceae (Kreft et al. 1999). Some plants containing rutin were used as repellents for preventing the stored products from insect infestation in China (Meng et al. 2003; Yu 2009). However, little is known about the behavioral response of *L. serricorne* adults to citronellal, citral, and rutin so far. Therefore, the aim of the present work was to evaluate the behavioral response of *L. serricorne* adults to citronellal, citral, and rutin.

## Methods

### Insects

Cultures of the cigarette beetle, *L. serricorne*, were maintained in the laboratory without exposure to any insecticide at the Institute of Stored Product Insects of Henan University of Technology. They were reared on a sterilized diet (wheatfeed/yeast, 95:5, w/w) at 27 ± 2 °C, 75 ± 5 % relative humidity, and a 12:12 light:dark photoperiod. Healthy, unsexed 3–5-day old adults were randomly chosen for bioassays.

### Preparation of the reagents

Citronellal is also called “rhodinal” or “3,7-dimethyloct-6-en-1-al”, and its molecular formula is C_10_H_18_O. Citral is also called “3,7-dimethyl-2,6-octadienal” or “lemonal”, and its molecular formula is C_10_H_16_O. Rutin’s molecular formula is C_27_H_30_O_16_. Citronellal, citral, and rutin of more than 96 % purity were obtained from Shanghai Jingchun Industry Ltd.

### Bioassay procedure

The behavioral response of *L. serricorne* adults to citronellal, citral, and rutin was evaluated by using the area preference method. Test areas consisted of Whatman No.1 filter paper cut in half (Ф12.5 cm). A series of citronellal or citral was respectively dissolved in ethanol (analytical purity) at the rate of 1:10, 1:50, 1:100 and 1:1000 (citronellal or citral: ethanol, v/v). Then the corresponding 500 µl solution was evenly applied on half-filter paper discs using a micropipette, respectively. The other half of the remaining filter paper was treated with 500 µl ethanol alone and used as a control. The filter papers were air-dried for about 5 min to evaporate the solvent completely and full discs were subsequently remade by attaching treated halves to untreated halves with clear adhesive tape. Each remade filter paper disc was tightly fixed onto the bottom of a petri dish (Ф12.5 cm) daubed with polytetrafluoroethylene (PTFE)on the inside wall to prevent the insects from escaping. For the rutin, the appropriate amount of powder was evenly spreaded on the half filter paper (Ф12.5 cm) according to the rate of 10, 30 and 90 g/m^2^. The other half of the remaining filter paper was untreated as a control. Twenty unsexed *L. serricorne* adults were then released at the center of the filter paper disc. The petri dishes were subsequently covered and kept in incubators at 27 ± 2 °C, 75 ± 5 % relative humidity, and a 12:12 light:dark photoperiod.

Each treatment was replicated three times and the number of insects present on the control (N_c_) and treated (N_t_) areas of the discs was recorded after 1, 2, 12 and 24 h, respectively.

Behavioral response values (BRV) were calculated as follows:

$${\text{BRV}} = [(N_{c} - N_{t} )/(N_{c} + N_{t} )]\;100\;\%$$

The positive behavioral response value (+) means repellent activity against the *L. serricorne* adults, and the negative behavioral response value (−) means attractive activity to the *L. serricorne* adults. The higher absolute value of BRV, the stronger repellent or attractive activity.

### Statistical analysis

The behavioral response value was determined and their absolute values of BRV were transformed to arcsine square-root values before subjecting to two-way analysis of variance (ANOVA) with BRV as response variable, and rate and exposure time as fixed effects. The mean behavioral response values were compared and separated by Scheffe’s test at p = 0.05 level. These analyses were performed using SPSS version 16.0 software.

## Results

Behavioral response of *L. serricorne* adults to citronellal significantly varied depending on tested rates (Tables 1, 2). The citronellal exhibited strong attractive activity at the low rate of 1:100 (v/v) during the whole exposure period, and the highest behavioral response value reached −88.89 %. However, it showed repellent activity at the high rate of 1:10 (v/v) (Table 1). *L. serricorne* adults had similar behavioral response to citral at different tested rates (Tables 3, 4). The citral also exhibited attractive activity at the low rate of 1:1000 (v/v) during the whole exposure period, however, it showed strong repellent activity at the high rates. Particularly, the citral could completely repel the *L. serricorne* adults at the rate of 1:50 (v/v) after 2 h exposure. Rutin had potent repellent activity against *L. serricorne* adults during the whole exposure period (Tables 5, 6), which significantly increased with increasing rates. The rutin could repel 100.00 % of the *L. serricorne* adults at the rate of 1:50 (v/v) after 12 h exposure.

**Table 1:** Behavioral response of *L. serricorne* adults to citronellal at the rate of 1:10, 1:50, 1:100 and 1:1000 (citronellal: ethanol, v/v) after 1, 2, 12 and 24 h exposure period, respectively

Rate (v:v)	Exposure time (h)			
	1	2	12	24
1:1000	−24.87 ± 3.38^c^	−53.97 ± 5.23^d^	−16.93 ± 1.58^b^	−77.78 ± 7.62^b^
1:100	−88.89 ± 10.99^d^	−77.27 ± 4.44^c^	−68.52 ± 3.38^c^	−81.48 ± 5.26^b^
1:50	5.86 ± 1.89^b^	8.44 ± 1.31^b^	22.75 ± 3.51^a^	27.02 ± 2.83^a^
1:10	52.38 ± 3.91^a^	52.38 ± 5.91^a^	25.11 ± 1.85^a^	44.44 ± 4.44^a^

Each datum represents the mean behavioral response value (±s.e.) of four replicates (*n* = 80). Means within a column followed by the same superscript letters are not significantly different at p < 0.05. The same as Tables 3 and 5

**Table 2:** Two-way ANOVA analysis for the behavioral response of *L. serricorne* adults to citronellal at the rate of 1:10, 1:50, 1:100 and 1:1000 (citronellal: ethanol, v/v) after 1, 2, 12 and 24 h exposure period, respectively

Fixed effects	*df*	F value	p value
Rate	3	4.458	0.010
Exposure time	3	0.465	0.709
Rate × exposure time	9	0.328	0.959
Error	32

**Table 3:** Behavioral response of *L.serricorne* adults to citral at the rate of 1:10, 1:50, 1:100 and 1:1000 (citral: ethanol, v/v) after 1, 2, 12 and 24 h exposure period, respectively

Rate (v:v)	Exposure time (h)			
	1	2	12	24
1:1000	−24.87 ± 3.39^c^	−53.97 ± 5.23^c^	−16.93 ± 1.32^d^	−77.78 ± 2.78^c^
1:100	30.16 ± 1.57^b^	51.85 ± 1.52^b^	9.99 ± 4.90^c^	37.61 ± 3.36^b^
1:50	96.30 ± 3.70^a^	100.00 ± 0.00^a^	73.68 ± 6.19^a^	67.72 ± 5.58^a^
1:10	82.08 ± 4.01^a^	74.67 ± 4.53^ab^	48.72 ± 3.71^b^	27.22 ± 1.48^b^

**Table 4:** Two-way ANOVA analysis for the behavioral response of *L. serricorne* adults to citral at the rate of 1:10, 1:50, 1:100 and 1:1000 (citral: ethanol, v/v) after 1, 2, 12 and 24 h exposure period, respectively

Fixed effects	*df*	F value	p value
Rate	3	15.649	0.000
Exposure time	3	1.132	0.351
Rate × exposure time	9	0.470	0.884
Error	32

**Table 5:** Behavioral response of *L.serricorne* adults to rutin at the rate of 10, 30 and 90 g/m^2^ after 1, 2, 12 and 24 h exposure period, respectively

Rate (g/m^2^)	Exposure time (h)			
	1	2	12	24
10.0	41.88 ± 4.27^a^	40.49 ± 1.60^b^	9.83 ± 3.14^b^	88.66 ± 3.58^a^
30.0	40.49 ± 1.60^a^	71.90 ± 5.23^a^	94.55 ± 3.21^a^	87.96 ± 7.23^a^
90.0	56.65 ± 5.93^a^	74.67 ± 4.53^a^	100.00 ± 0.00^a^	100.00 ± 0.00^a^

**Table 6:** Two-way ANOVA analysis for the behavioral response of *L. serricorne* adults to rutin at the rate of 10, 30 and 90 g/m^2^ after 1, 2, 12 and 24 h exposure period, respectively

Fixed effects	*df*	F value	p value
Rate	2	13.652	0.000
Exposure time	3	9.614	0.000
Rate × exposure time	6	4.437	0.004
Error	24		

## Discussion

The behavioral response of insects to compounds depends on insect species, developmental stages, strains, rates, compound components, application methods and special environmental factors (Kanzaki 1996; Watson and Barson 1996; Fields et al. 2001). Some compounds which can significantly attract insects, particularly sex pheromones, have been developed as attractants for monitoring programs (Burkholder and Ma 1985; Trematerra 2012), or as attracticides (Nansen and Phillips 2004). Some compounds which can significantly repel insects have been developed as repellants for insect disinfestation, especially in insect-resistant packaging. Many materials such as synthetic pyrethroids, citronella, natural botanical antifeedants, (E)-2-hexenal, silicagel, and protein-enriched pea flour have been verified to effectively protect packaging materials against stored product insects and some of them are being applied on packaging materials for their effectiveness against insect penetration (Bloszyk et al. 1990; Wong et al. 2005; Germinara et al. 2012a, b).

The citronellal, citral, and rutin are safe, because they extensively exist in the citrus fruits and other fruit plants which are usually used as cosmetics, flavoring agents and traditional herbal medicines (Onawunmi 1989; Li et al. 2015; Randazzo et al. 2016; Stoldt et al. 2016). The present research results demonstrates that citronellal and citral can greatly attract *L. serricorne* adults at lower rates, and repel *L. serricorne* adults at higher rates. Moreover, rutin significantly repels *L. serricorne* adults. Provided with a proper formulation, rate, and reasonable application strategy, citronella, citral, and rutin may be used to effectively prevent *L. serricorne* infestations. Meanwhile, the effectiveness of the citronellal, citral, and rutin against *L. serricorne* larvae deserves to be further investigated in the future, because the *L. serricorne* larvae usually results in the most serious loss due to their mass feeding and harmful metabolite (Mahroof and Phillips 2014). The effect of *L. serricorne* adults and larvae long-term contacting the citronellal, citral, and rutin also needs to be researched, because this will affect the behavioral response of *L. serricorne* adults and larvae.

## Conclusions

In summary, citronellal, citral, and rutin have great potential as repellents or attractants for managing *L. serricorne* adults at suitable formulations and rates in practice, and a proper formulation, rate, and reasonable application strategy for each compound deserves to be further investigated as soon as possible.
